# Increased expression of neurotensin in high grade serous ovarian carcinoma with evidence of serous tubal intraepithelial carcinoma

**DOI:** 10.1002/path.5264

**Published:** 2019-05-14

**Authors:** Eric J Norris, Qing Zhang, Wendell D Jones, Darla DeStephanis, Ashley P Sutker, Chad A Livasy, Ram N Ganapathi, David L Tait, Mahrukh K Ganapathi

**Affiliations:** ^1^ Levine Cancer Institute, Atrium Health Charlotte NC USA; ^2^ Department of Bioinformatics and Clinical Systems, Q^2^ Solutions – EA Genomics Morrisville NC USA; ^3^ Carolinas Pathology Group Charlotte NC USA

**Keywords:** ovarian cancer, serous tubal intraepithelial carcinoma, fallopian tube, neurotensin

## Abstract

High grade serous ovarian carcinoma (HGSC) without identifiable serous tubal intraepithelial carcinoma (STIC) within the fallopian tube (FT) occurs in approximately 50% of patients. The objective of this study was to use a multisite tumor sampling approach to study HGSC with and without STIC. RNAseq analysis of HGSC samples collected from multiple sites e.g. ovary, FT and peritoneum, revealed moderate levels of intrapatient heterogeneity in gene expression that could influence molecular profiles. Mixed‐model ANOVA analysis of gene expression in tumor samples from patients with multiple tumor sites (*n* = 13) and patients with a single site tumor sample (*n* = 11) to compare HGSC‐STIC to HGSC‐NOSTIC identified neurotensin (*NTS*) as significantly higher (> two‐fold change, False Discovery Rate (FDR) < 0.10) in HGSC‐STIC. This data was validated using publicly available RNA‐Seq datasets. Concordance between higher *NTS* gene expression and NTS peptide levels in HGSC‐STIC samples was demonstrated by immunohistochemistry. To determine the role of NTS in HGSC, five ovarian cancer (OvCa) cell lines were screened for expression of NTS and its receptors, NTSR1 and NTSR3. Increased expression of NTS and NSTR1 was observed in several of the OvCa cells, whereas the NTSR3 receptor was lower in all OvCa cells, compared to immortalized FT epithelial cells. Treatment with NTSR1 inhibitor (SR48692) decreased cell proliferation, but increased cell migration in OvCa cells. The effects of SR48692 were receptor mediated, since transient RNAi knockdown of NTSR1 mimicked the migratory effects and knockdown of NTSR3 mimicked the anti‐proliferative effects. Further, knockdown of NTSR1 or NTSR3 was associated with acquisition of distinct morphological phenotypes, epithelial or mesenchymal, respectively. Taken together, our results reveal a difference in a biologically active pathway between HGSC with and without STIC. Furthermore, we identify neurotensin signaling as an important pathway involved in cell proliferation and epithelial–mesenchymal transition in HGSC‐STIC which warrants further study as a potential therapeutic target. © 2019 The Authors. *The Journal of Pathology* published by John Wiley & Sons Ltd on behalf of Pathological Society of Great Britain and Ireland.

## Introduction

High grade serous ovarian carcinoma (HGSC) is the most common and deadly subtype of epithelial ovarian cancer (EOC) and accounts for a disproportionate high number of cancer related deaths in the United States 1. Approximately 70% of patients with HGSC have advanced stage disease [International Federation of Gynecology and Obstetrics (FIGO) stages III and IV] at the time of diagnosis. Clinical management of HGSC includes cytoreductive surgery and adjuvant chemotherapy with a platinum/taxane based regime. While most patients respond to first‐line chemotherapy, a majority experience tumor recurrence often with chemo‐resistant disease 2, 3. As a result, prognosis for HGSC is dismal and there remains an urgent clinical need to better understand the disease.

Extensive pathological review of fallopian tubes (FTs) from women with HGSC and women predisposed to ovarian cancer (i.e. *BRCA1/2* mutation) undergoing prophylactic bilateral salpingo‐oophorectomy has elucidated the initiating events of HGSC 4. It is now known that HGSC originates from precursor lesions, termed serous tubal intraepithelial carcinoma (STIC), located in the fimbriated end of the FT in most patients. However, an important caveat regarding the FT‐origin theory involves the inability to identify a STIC in 39–89% of HGSC patients 5, 6. Technical discrepancies aside, recent evidence suggests that the pathogenesis of HGSC without STIC (NOSTIC) may be biologically distinct from HGSC with co‐existing STIC 7. Termed ‘precursor escape’, the model proposes that HGSC develops from early serous proliferations that are shed from the FT mucosa prior to malignant transformation to STIC 6. Alternatively, it remains possible that ovarian surface epithelial (OSE) cells undergo Müllerian metaplasia and malignant transformation without involving the FT 8.

A major obstacle in molecular profiling of HGSC is the high degree of interpatient heterogeneity, existing between tumors from different patients, and intrapatient tumor heterogeneity, existing between synchronous, spatially discrete tumors 9. In particular, intrapatient heterogeneity negatively influences molecular profiling in various cancers leading to the suggestion that analyzing multiple tumors from a single patient may improve molecular profiling studies 10, 11, 12, 13. Thus, the aim of the present study was to use a multisite tumor sampling approach to compare the molecular profiles between HGSC with and without STIC. To our knowledge, this is the first study to use RNAseq analysis to demonstrate that multisite tumor sampling from defined anatomical sites in individual patients with HGSC can establish molecular differences between HGSC‐STIC and HGSC‐NOSTIC and the identification of neurotensin (NTS) as a key signaling entity.

## Materials and methods

### Patient sample collection

All patients were consented according to protocols established by the institutional review board at Atrium Health in Charlotte, NC, USA. Patient samples were obtained at the time of primary tumor debulking surgery. Samples were collected from the right and left ovary (OV), right and left FT, and one metastatic implant from within the peritoneum and placed in 7.5 ml of RNAlater® (Sigma‐Aldrich, St. Louis, MO, USA) overnight at 4° C. The anatomical site and tumor involvement of each sample was confirmed by a board‐certified pathologist with expertise in gynecologic malignancies, prior to analysis.

### Assessment of STIC

Samples were assigned as either HGSC‐STIC or HGSC‐NOSTIC using the criteria for primary site assignment of OV, FT or primary peritoneal HGSC at our institution. The criteria are as follows: (1) The tumor primary site is assigned as FT in the presence of STIC or invasive mucosal carcinoma involving the FT (HGSC‐STIC); (2) the primary site is assigned as OV for cases demonstrating ovarian mass without identified STIC (using SEE‐FIM protocol) or invasive mucosal carcinoma in either tube (HGSC‐NOSTIC); or (3) primary site is classified as peritoneal (P‐HGSC) when both tubes and ovaries are grossly normal with no more than 5 mm of ovarian surface stromal involvement by tumor and no STIC (using SEE‐FIM protocol).

### Cell lines and culture

Immortalized FT epithelium cells (FTE 237, FTE 240, and FTE 246) were the gift of Dr. Ronny Drapkin at the University of Pennsylvania, Perelman School of Medicine, Philadelphia, PA, USA. Immortalized human ovarian surface epithelium HOSE cells were the gift of Dr. H. Katabuchi at Kumamoto University in Kumamoto, Japan and IOSE 385 and IOSE 397 were procured from the Canadian Ovarian Tissue Bank in Vancouver, Canada. Ovarian cancer cell lines (OvCa) PEO1 and OVCAR5 were the gift of Dr. Thomas Hamilton, Fox Chase Cancer Center, Philadelphia, PA, USA. OVCAR3 was obtained from American Type Tissue Collection (ATCC, Manassas, VA, USA). PEO4 cell line was obtained from Sigma‐Aldrich. UPN‐251 cells were obtained from Bristol Myers Squibb (New York, NY, USA). In accordance to journal guidelines, all cell lines were authenticated by STR analysis if historical STR profiles were available, and none of the cell lines are listed in the International Cell Line Authentication Committee database (https://iclac.org/). Of note, we have included OVCAR5 cells in our study which were originally isolated from a patient with ovarian cancer and remain widely used in the literature as an in vitro model of ovarian cancer *per se* (https://web.expasy.org/cellosaurus/CVCL_1628). However, it should be noted for proper context that recent transcriptomic profiling has demonstrated a possible nonovarian origin for this cell line. In all cases, OVCAR5 data is presented alongside two different cell lines verified to be derived from EOCs. All cells were maintained at 37 °C in a humidified 5% CO_2_ plus 95% air atmosphere. A description of culture conditions is available in the supplementary material, Supplementary materials and methods.

### RNAsequencing and bioinformatics

RNA was isolated from tumor samples using the Direct‐zol RNA Mini‐Prep kit (Zymo Research, Irvine, CA, USA) following the manufacturer's protocol. NextGen RNA‐Seq analysis was performed by Q^2^ Solutions (Morrisville, NC, USA). In brief, 100 ng of RNA was subjected to ribosomal RNA (rRNA) depletion, reverse transcribed into cDNA, then amplified using PCR. Final libraries were sequenced to at least 45 M paired reads (most in 45–60 M range). The pipeline RNAv9 (EA‐Quintiles) was used to analyze RNA‐Seq data. The RSEM v1.2.0 program (available at http://deweylab.github.io/RSEM/), RSEM‐calculate‐expression, was run with parameters optimized for Illumina 50 × 50 paired‐end sequencing (Illumina, San Diego, CA, USA). The University of California, Santa Cruz (UCSC) known gene transcriptome was used for sequence alignment. For across sample analysis, upper‐quartile normalization of the read counts was performed. For batches of cancer samples processed separately over time, a subset of three samples had distinct libraries created and sequenced in each batch. Batch‐related effects were estimated and removed gene by gene for all target samples based on the effects estimated via ANOVA from the replicate signal (log_2_ of normalized counts) of these three reference samples. A detailed description of methods employed in bioinformatic analysis can be found in the supplementary material, supplementary materials and methods. The normalized gene level expression (log_2_) values for each sample analyzed in this study are available in supplementary material, Table S3.

### Hierarchical clustering and differential gene expression

Gene‐level expression of the 1500 most variant genes was centered using *Z*‐scores and then hierarchical clustering analysis using a distance measure of ‘one minus the Pearson correlation’ with complete linkage of samples and genes was performed using the Morpheus program (https://software.broadinstitute.org/morpheus) available online from the Broad Institute (Massachusetts Institute of Technology, Cambridge, MA, USA). Genes with mean expression values in the lowest quartile were excluded from analysis. To investigate an association between the presence of STIC and published TCGA molecular subclasses, tumor samples were clustered using a published 100‐gene signature which clusters tumor samples with similar expression profiles representative of one of the TCGA subtypes ( ‘differentiated’, ‘immunoreactive’, ‘mesenchymal,’ or ‘proliferative’) 14. For differential gene expression analysis, a linear mixed model was estimated including presence of STIC (HGSC‐STIC, HGSC‐NOSTIC) and tumor anatomic location – FT, OV, metastasis (Met) – as fixed factors. Subject was included in the model as a random factor to account for repeated measures on the same subject. Maximum likelihood estimation was utilized, and statistical testing was performed using the Satterthwaite approximation for denominator degrees of freedom. Multiplicity adjustments for the pairwise comparisons among the three anatomic locations were conducted using the Tukey–Kramer *post hoc* method. Benjamini–Hochburg method was used to adjust for multiple‐hypothesis testing. False Discovery Rate (FDR) <0.10 was used to identify differentially expressed genes. For validation, expression of identified genes was compared between HGSC‐STIC and HGSC‐NOSTIC in two publicly available datasets, Ducie *et al* 14 and TCGA Ovarian Cancer dataset 15 using a one‐tailed Student's *t* test. *p* < 0.05 was considered statistically significant.

### Immunohistochemistry

Frozen sections were collected on PLUS slides (VWR, Radnor, PA, USA), fixed in formaldehyde and incubated overnight with a commercially available validated primary antibody against NTS (1:1000, #AB5496, EMD Millipore, Burlington, MA, USA). The percentage of tumor cells staining positive for NTS was assessed independently by a pathologist and a research scientist in a blinded manner and scored as negative (<1% positive staining), low (1–10% positive staining), or high (>10% positive staining). Sections of small intestine stained with NTS antibody served as a positive control (see supplementary material, Figure S1).

### Cell migration assay

Collective cell migration was assessed using the Wound Healing Assay as described previously 16. In brief, cells were seeded into 12‐well tissue culture treated plates and allowed to attach overnight. Wells were washed with serum free media and a scratch was made to the confluent layer using a 200 μl micropipette tip. Scratches were visualized immediately after scratch and 18 h later using an inverted IX73 Olympus microscope, automated microscope stage (Prior Scientific, Cambridge, UK) and CellSens Software (Olympus, Tokyo, Japan). The average width of the scratch was calculated as the total area divided by the length of the scratch. All treatments were performed in triplicate and each experiment was performed at least three times.

### Transient RNAi transfections

OVCAR3, OVCAR5, and UPN‐251 cells were seeded into either 6‐well or 12‐well plates with Roswell Park Memorial Institute (RPMI) 1640 containing 10% FBS and allowed to attach overnight. On the following day, media was replaced with Opti‐mem media (Thermo Fisher Scientific, Waltham, MA, USA) containing 5 μl/ml Lipofectamine RNAi Max reagent (Thermo Fisher Scientific) and 20 nm Silencer® Select RNAi targeting either *NTSR1* (assay ID 143659), *NTSR3* (assay ID 142525) or scrambled nontargeting control (Thermo Fisher Scientific). For proliferation studies, RNAi containing media was replaced with RPMI 1640 plus 1% FBS following 16 h incubation and cell proliferation was assessed after an additional 96 h. For the wound healing assay, a scratch was made prior to replacing RNAi containing media with serum free RPMI 1640 and migration was assessed after 18 h.

### RT‐qPCR, western blotting, and ELISA

Detailed protocols are presented in supplementary material, Supplementary materials and methods.

## Results

Tumor samples were collected from patients with HGSC undergoing primary tumor debulking surgery. Patients were excluded from analysis due to ambiguous site of tumor origin, stages 1 or 2 disease, diagnosis of primary peritoneal HGSC, history of neo‐adjuvant chemotherapy, or insufficient RNA yield. In total, 24 patients were used for RNAseq comparison between HGSC‐STIC (*n* = 9) and HGSC‐NOSTIC (*n* = 15). Clinical characteristics were similar between the two groups (see supplementary material, Table S1). Tumor samples from multiple anatomical sites were collected from 13 of the 24 patients (see supplementary material, Table S2), which included 4/9 (44%) HGSC‐STIC and 9/15 (60%) HGSC‐NOSTIC patients.

**Figure 1:**
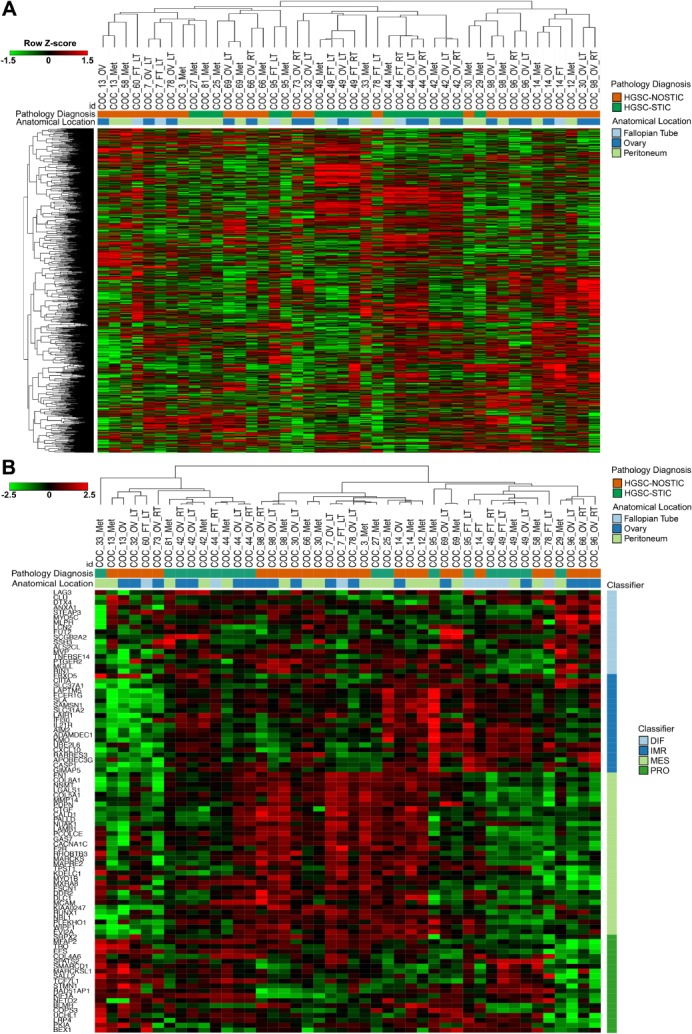
Unsupervised and supervised hierarchical clustering of HGSC tumor samples. (A) Expression levels of the top 1500 most variant genes from all tumor samples were used for unsupervised hierarchical clustering analysis using ‘One Minus Pearson Correlation’ with complete linkage. (B) Supervised clustering of paired and unpaired tumor samples using a 100‐gene signature for TCGA molecular subclassification. Diagnosis (HGSC‐STIC versus HGSC‐NOSTIC), anatomical location and presumed TCGA classification (differentiated, immunoreactive, mesenchymal, proliferative) are color coded as indicated. Heatmaps were prepared using log_2_ read counts and row *Z*‐score centering for each gene. In total, the 44 samples analyzed included 33 paired samples from 13 patients and 11 unpaired samples from 11 patients.

Unsupervised hierarchical agglomerative clustering of tumor samples did not cluster into clear HGSC‐STIC or HGSC‐NOSTIC groups (Figure 1A). Rather, tumor samples from different anatomical sites tended to cluster with samples from the same patient. Of note, in three patients (COC_30, COC_78, COC_98) at least one sample clustered more closely with tumors from other patients than the other anatomical sites in the same patient, suggesting a high degree of intrapatient heterogeneity in some patients. A similarity matrix constructed using the Pearson correlation coefficient between samples from patients with multiple tumor sites confirmed a lower correlation (range 0.1–0.56) between interpatient samples compared to intrapatient samples (range 0.31–0.90) indicating a high degree of interpatient heterogeneity (see supplementary material, Figure S2).

To determine if the presence of STIC was associated with one of the four HGSC molecular subtypes identified in the TCGA ovarian cancer dataset 17, tumor samples were clustered using a published 100‐gene score signature 14. No obvious grouping was observed between HGSC‐STIC or HGSC‐NOSTIC and any of the TCGA subgroups (Figure 1B). Interestingly, for this 100‐gene signature, tumor samples from the same patient clustered together in only 6 of the 13 patients with multiple tumor site samples (Figure 1B) compared to 10 out of 13 when the most variant genes were used (Figure 1A). Moreover, in three patients (COC_14, COC_66, COC_78) tumor samples from the same patient appeared to cluster with samples associated with different TCGA subtypes.

Using a linear mixed‐model ANOVA to account for patient effects, four genes (*IMPACT, NTS, LOC645591, TMEM168*) were identified as differentially expressed between HGSC‐STIC and HGSC‐NOSTIC after adjustment for multiple testing (FDR < 0.10) (Table 1). Of these, *NTS* (9.5‐fold, *q* = 0.006) and *LOC645591* (4.0‐fold, *q* = 0.05) demonstrated greater than two‐fold increase in HGSC‐STIC. For validation, we queried a published dataset that compared gene expression profiles of HGSC with and without STIC in 85 patients 14. Both *NTS* (*p* = 0.037) and *LOC645591* (*p* = 0.045) were higher in HGSC associated with STIC compared to HGSC without STIC in the validation dataset. For additional validation, we reviewed pathology reports from the TCGA ovarian cancer dataset for evidence of STIC to classify patients as either HGSC‐STIC or HGSC‐NOSTIC (see supplementary material, Supplementary materials and methods 15). We identified a cohort of 19 patients classified as HGSC‐STIC and 12 as HGSC‐NOSTIC that fit the criteria in our study. Analysis of this dataset confirmed increased expression of *NTS* in the HGSC‐STIC group (*p* = 0.02). Since the TCGA RNA‐Seq methods used a nonstrand specific protocol, *LOC645591* expression could not be validated as the measurement of sense and antisense transcripts are confounded.

**Table 1:** Differentially expressed genes between HGSC‐STIC and HGSC‐NOSTIC

Gene ID	Fold‐change	*P* value	*q* value	Fold‐change 14	*P* value 14	*P* value (TCGA)
*NTS*	9.48	4.94E−07	5.92E−03	1.93	0.037	0.020
*IMPACT*	−1.54	3.95E−07	5.92E−03	1.50	NS	N/A
*LOC645591*	3.98	6.35E−06	5.08E−02	1.66	0.045	N/A
*TMEM168*	−1.58	9.87E−06	5.92E−02	1.10	NS	N/A

A linear mixed model ANOVA was utilized to identify differentially expressed genes (FDR < 0.10) between HGSC‐STIC and HGSC‐NOSTIC. The fold change, unadjusted *P* value, and adjusted *P* value (Benjamini–Hochberg method) for the four differentially expressed genes identified are listed. Expression of the differentially expressed genes with greater than two‐fold differences between HGSC‐STIC and HSGC‐NOSTIC was assessed in two independent publicly available datasets, 14 and TCGA ovarian cancer dataset 15. A one‐tailed Student's *t*‐test was used to test for significant differences in the validation datasets. *p* < 0.05 was considered statistically significant.

NS, nonsignificant; N/A not available.

**Figure 2:**
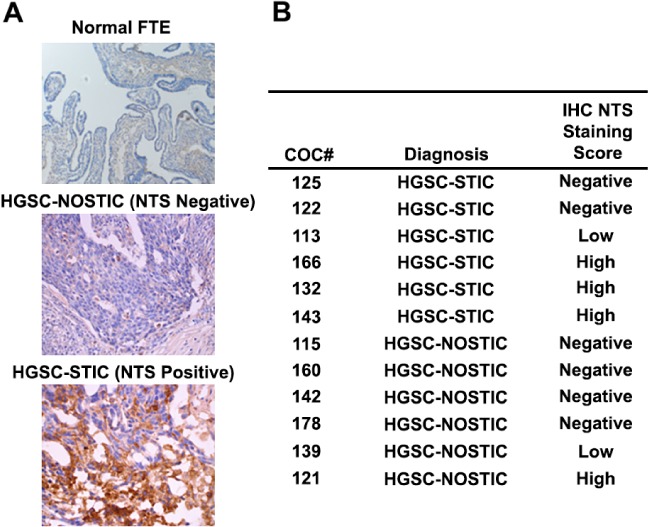
Expression of NTS in HGSC tumor samples and normal FT. NTS expression in HGSC‐STIC (*n* = 6) and HGSC‐NOSTIC (*n* = 6) samples was assessed by immunohistochemistry in frozen tumor sections using a commercially available validated antibody. (A) Representative photomicrographs showing normal FT with negative staining for NTS, HGSC‐STIC with positive staining for NTS, and HGSC‐NOSTIC with negative staining for NTS. (B) Positive staining was assessed in a blinded manner by two independent researchers. Tumor samples were scored by the percentage of tumor cells staining positive for NTS irrespective of staining intensity. Tumor samples were scored as negative with <1% positively stained cells, low with 1–10% positive stained cells, and high if >10% of tumor cells stained positively for NTS.

Since *NTS* was the only coding gene identified, we performed immunohistochemical staining of a separate HGSC patient cohort HGSC‐STIC (*n* = 6), HGSC‐NOSTIC (*n* = 6) to examine concordance between *NTS* gene and NTS peptide expression (Figure 2). NTS staining was observed in 4/6 (67%) HGSC‐STIC samples and in 2/6 (33%) HGSC‐NOSTIC samples. Moreover, of the four tumor samples with ‘high’ NTS staining, three were HGSC‐STIC samples. Because circulating levels of NTS are reported to be elevated in colorectal carcinoma 18, we tested serum NTS levels in patients with HGSC. While NTS levels were elevated in patients with HGSC compared to healthy controls (*p* = 0.0165, see supplementary material, Figure S3), no differences were observed between HGSC‐STIC and HGSC‐NOSTIC (data not shown).

**Figure 3:**
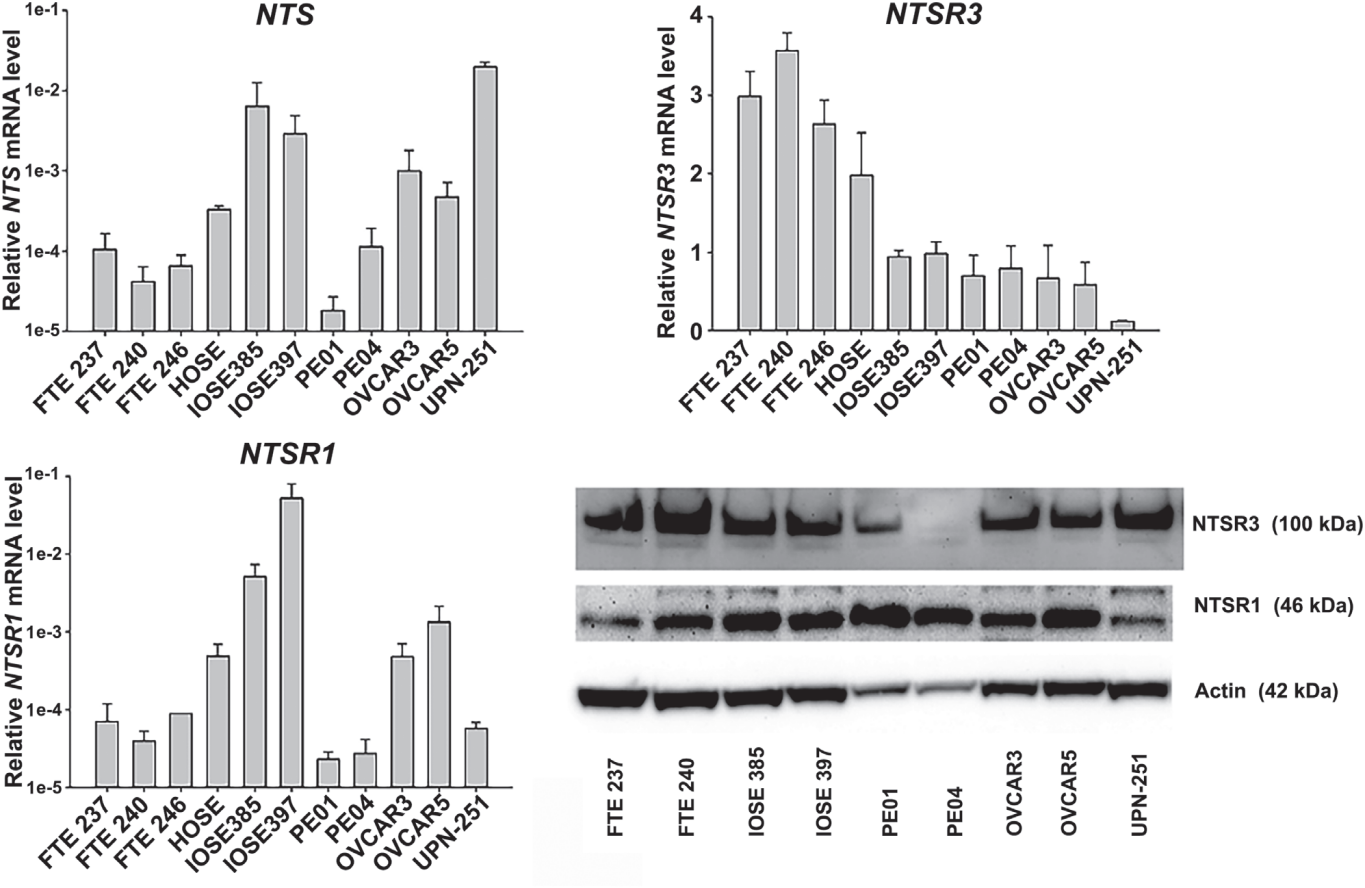
Expression of NTS and its receptors in FTE, OSE, and OvCa cell lines‐ Expression of NTS, NTSR1, and NTSR3 was assessed in immortalized FTE cells, immortalized OSE cells, and OvCa cells by RT‐qPCR using validated Taqman probes. Target gene mRNA expression was normalized to *UBTF*, an invariant reference gene, and reported as relative mRNA expression. Protein expression of NTSR1 and NTSR3 was assessed by western blotting using commercially available antibodies. β‐Actin served as a loading control. Immunoblots are representative of three separate experiments.

To investigate the function of NTS signaling in HGSC, we first assessed the expression of *NTS* and its receptors (*NTSR1*, *NTSR2*, *NTSR3*) in a panel of FTE, OSE and OvCa cell lines (Figure 3). *NTS* expression was low (Ct ≥ 37 cycles), but detectable by RT‐qPCR in all three FTE cells and 2/5 OvCa cell lines (PEO1, PEO4). Moderate *NTS* expression was observed in all three OSE cell lines and the remaining OvCa cell lines (OVCAR3, OVCAR5, UPN‐251). Western blotting revealed higher expression of NTSR1 protein in 2/3 OSE and, interestingly, 4/5 OvCa cell lines compared to normal FTE. NTSR3 expression was highest in FTE cells compared to OSE and OvCa cell lines.

**Figure 4:**
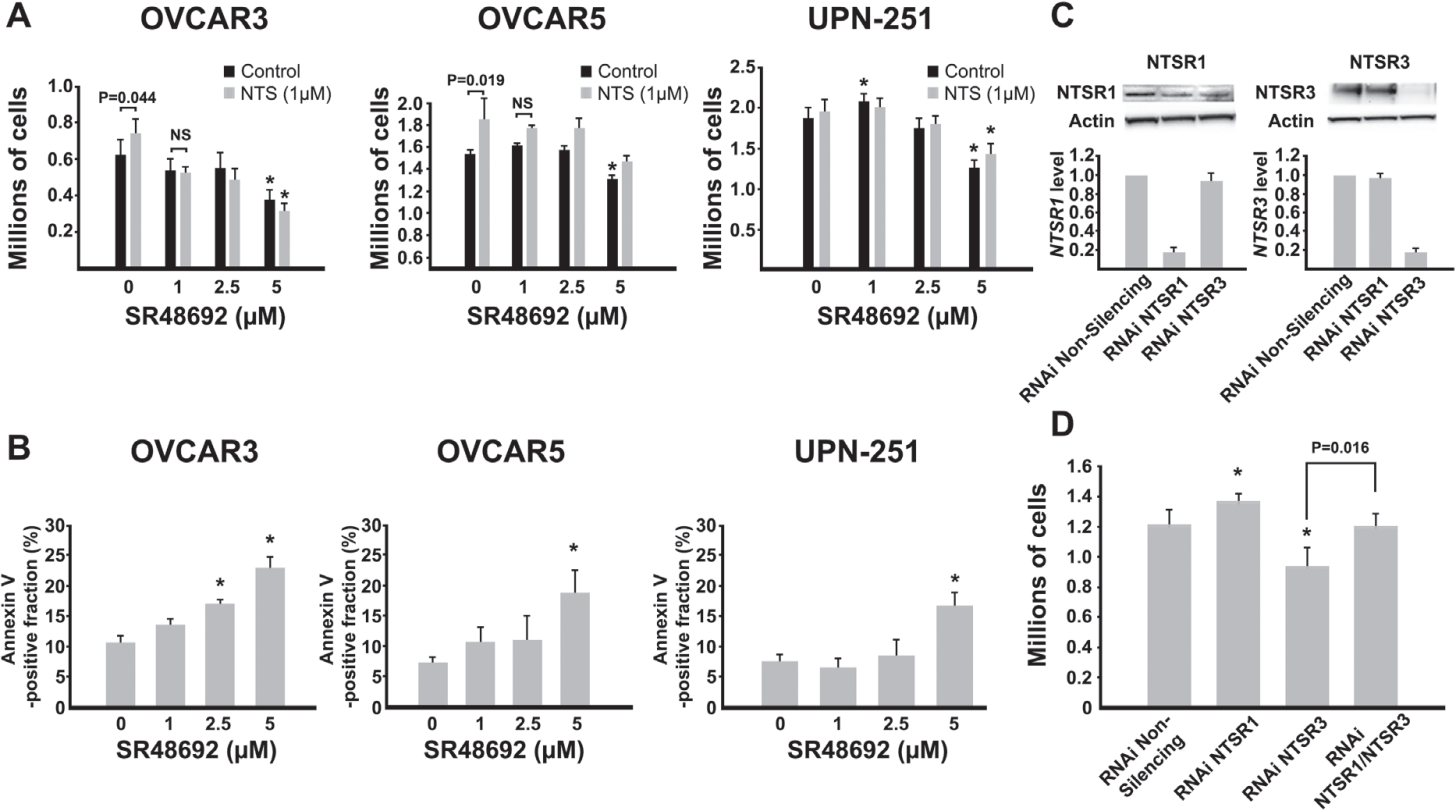
Effect of NTS and NTSR1 inhibitor SR48692 in OvCa cell lines. (A) NTS‐expressing OvCa cells (OVCAR3, OVCAR5, UPN‐251) were treated without (black bars) or with (grey bars) exogenous NTS (1 μm) and cell proliferation was assessed at the end of 120 h of culture. Each experiment was performed at least three times and a two‐way ANOVA with Student–Newman–Keuls *post hoc* test was used to test for significant differences. *p < 0.05 compared to control. (B) OVCAR3, OVCAR5, and UPN‐251 cells were treated with increasing concentrations of SR48692. At the end of 120 h, apoptosis (Annexin V staining) was assessed. A one‐way ANOVA with Dunnett's *post hoc* test was used to determine statistical significance. *p < 0.05 compared to control. (C) OVCAR3 cells were treated with Silencer Select® RNAi targeting *NTSR1* or *NTSR3* or nonsilencing siRNA. NTSR1 and NTSR3 mRNA and protein expression was determined at 72 h by RT‐qPCR and western blotting, respectively. (D) Cell proliferation was assessed after 120 h. A one‐way ANOVA with Student–Newman–Keuls *post hoc* test was used to determine statistical significance. *p < 0.05 as indicated.

NTS‐expressing OVCAR3, OVCAR5, and UPN‐251 cells were used to investigate the NTS signaling pathway in HGSC. Compared to untreated cells (black bars), treatment with exogenous NTS (1 μm, grey bars) for 120 h increased proliferation in OVCAR3 (*p* = 0.046) and OVCAR5 (*p* = 0.019) cells, but not UPN‐251 cells which expressed the highest level of *NTS* (Figure 4A). The stimulatory effect of NTS in OVCAR3 and OVCAR5 cells was attenuated by cotreatment with the specific NTSR1 inhibitor SR48692 (1 μm). Interestingly, a biphasic effect of SR48692 was observed in UPN‐251 cells because low concentrations were associated with a modest, but statistically significant increase in cell number. High concentrations of SR48692 inhibited cell growth and induced apoptosis in all lines tested (Figure 4A,B). The effects of SR48692 can occur directly through NTSR1 or indirectly through NTSR3 via an interaction with NTSR1 in cells that express both receptors 19, 20. A targeted transient RNAi knockdown approach was employed in OVCAR3 cells (Figure 4C) to identify which receptors were responsible for the growth inhibitory effects of SR48692. RNAi knockdown of NTSR1 stimulated, whereas RNAi knockdown of NTSR3 inhibited cell proliferation suggesting that the growth inhibitory effect of SR48692 is mediated via NTSR3 receptor (Figure 4D). Interestingly, co‐transfection with both RNAi‐*NTSR1* and RNAi‐*NTSR3* had no effect on cell growth compared to control.

**Figure 5:**
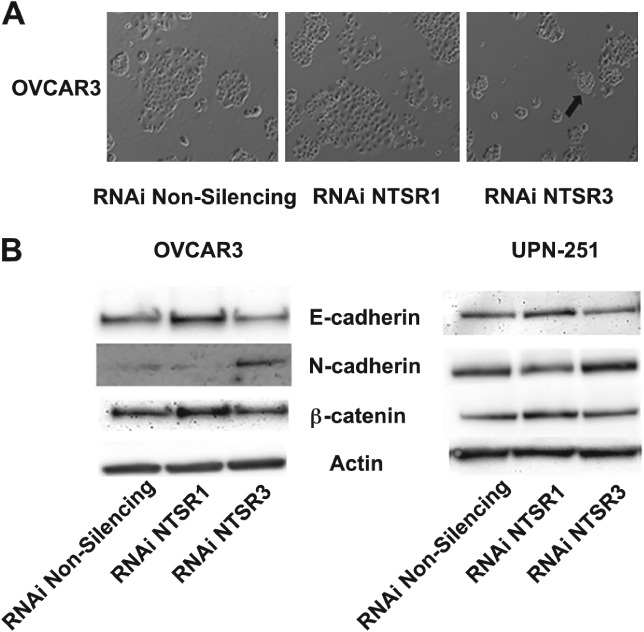
Transient knockdown of NTSR1 and NTSR3 modulates epithelial to mesenchymal transition in OvCa cells. (A) Morphologic changes in OVCAR3 cells transfected with RNAi against NTSR1 and NTSR3 for 96 h. Black arrow indicates a loosely adherent, spontaneous spheroid budding from the monolayer. Micrographs are representative of at least three experiments. (B) Immunoblot analysis of cell lysates from OVCAR3 and UPN‐251 cells treated for 96 h with RNAi targeting NTSR1 or NTSR3 probed with antibodies against markers of epithelial (E‐cadherin, β‐catenin) and mesenchymal (N‐cadherin) cells. Immunoblots are representative of three separate experiments. β‐Actin served as a loading control.

While conducting the RNAi experiments, an unexpected phenotypic shift in the cells was observed that suggested NTS signaling may modulate epithelial to mesenchymal transition. For example, in OVCAR3 cells, NTSR1 knockdown was associated with large clusters of cells, whereas NTSR3 knockdown was associated with smaller clusters of irregularly sized cells and the appearance of spontaneously forming spheroids which were loosely adherent to the flask (Figure 5A). Accordingly, NTSR1 knockdown was associated with increased expression of the epithelial marker E‐cadherin and β‐catenin, whereas NTSR3 knockdown was associated with increased expression of the mesenchymal marker N‐cadherin in OVCAR3 cells as well as UPN‐251 cells (Figure 5B). While no change in cadherin expression was observed in OVCAR5 cells, NTSR1 knockdown was associated with an induction of the EMT associated transcription factor SNAIL (see supplementary material, Figure S4).

**Figure 6:**
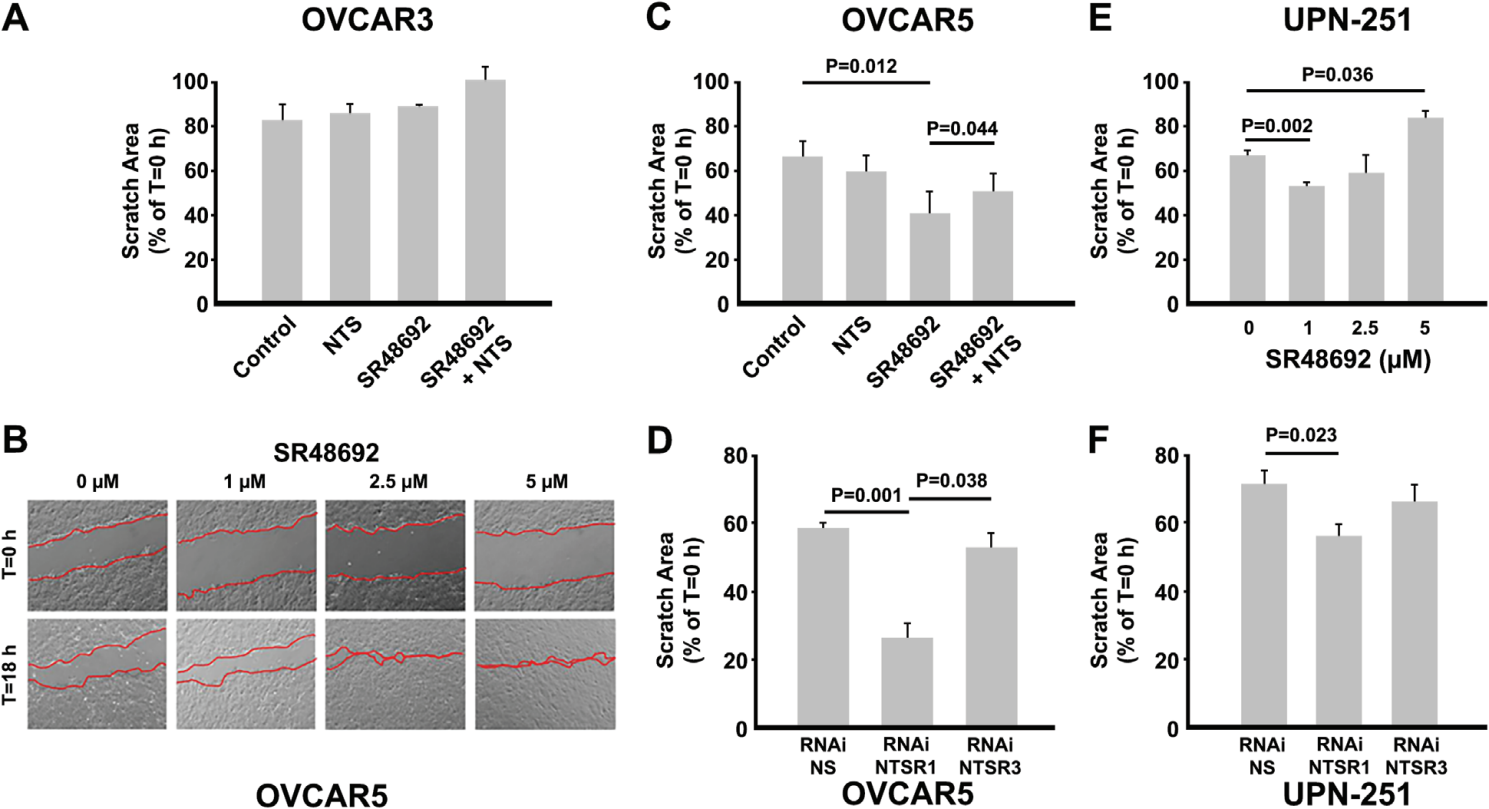
Effect of pharmacologic inhibition of NTSR1 or downregulation of *NTSR1* or *NTSR3* mRNA on collective cell migration. (A) Wound healing assays in OVCAR3 cells were performed to assess the effect of NTS (1 μm) and SR48692 (1 μm) on collective cell migration. (B,C) Dose dependent increase in collective cell migration by SR48692 in OVCAR5 cells, which is attenuated by cotreatment with NTS. (D) OVCAR5 cells transfected with RNAi targeting NTSR1 or NTSR2 demonstrate increased collective cell migration. RNAi knockdown of NTSR3 has no effect. (E,F) Effect of pharmacologic inhibition or RNAi knockdown of NTSR1 on collective cell migration was assessed in UPN‐251 cells. Each experiment was performed a minimum of three times and a two‐ or one‐way ANOVA with Student–Newman–Keuls *post hoc* test was used to detect statistical significance. *p < 0.05 where indicated. Micrographs are representative of three individual experiments with treatments performed in triplicate.

Because NTS modulates cell migration in various types of cancer 21, wound healing assays were performed on cells treated with either NTS (1 μm) or SR48692 (1 μm) to assess the effect of NTS signaling on cell migration in HGSC. In OVCAR3 cells, treatment with neither NTS nor SR48692 affected cell migration and this is consistent with studies demonstrating low migratory capacity of OVCAR3 cells (Figure 6A) 22. However, in the highly migratory OVCAR5 cells SR48692 caused a dose‐dependent increase in collective cell migration which was attenuated by combined treatment with NTS (Figure 6B,C). RNAi knockdown of NTSR1 was associated with increased collective cell migration mimicking the effect of SR48692 (Figure 6D). No effect was observed when NTSR3 was targeted. Similar results were observed in UPN‐251 cells treated with SR48692 or NTSR1 RNAi (Figure 6E,F). Of note, similar to its effect on cell proliferation, a biphasic response to SR48692 was observed in UPN‐251 cells. Since epithelial‐like cells tend to migrate via collective cell migration 23, this finding further supports an epithelial like phenotype in NTSR1 RNAi treated cells.

## Discussion

This study is the first to report differences in gene expression between HGSC‐STIC and HGSC‐NOSTIC. Because molecular profiling studies in HGSC are hindered by intrapatient heterogeneity 9, 24, we sought to analyze multisite tumor samples from each patient. In agreement with previous reports, we found that global expression profiles of tumors do not differentiate between HGSC‐STIC and HGSC‐NOSTIC nor is there any association between HGSC‐STIC and TCGA molecular subtypes 14. Moreover, our analysis suggests that tumor samples taken from multiple sites from a single patient may exhibit molecular profiles that associate with different representative TCGA molecular subtype groupings. The implications of this finding are two‐fold. First, gene expression profiles from a single HGSC tumor sample are subject to random sampling bias and might not be representative of tumor at different anatomical sites 25. Second, as others have suggested, molecular profiling may require multiple tumor replicates from each patient to account for accurate estimates of gene expression 26.

Indirect evidence from studies modeling normal FTE and OSE have shown that most HGSC tumor samples resemble FT epithelial cells 27, 28, 29. Moreover, direct comparison of HGSC‐STIC to HGSC‐NOSTIC have found few differences suggesting the two groups are one entity 14, 30. In the most comprehensive study to date, Ducie and colleagues used an integrative Next‐Generation sequencing approach that was specifically designed with sufficient power so that acceptance of the null hypothesis was evidence that HGSC with and without STIC are not unique entities 14. Accordingly, after adjusting for multiple hypotheses testing, no differentially expressed genes were found (*q* < 0.10). In the same study, 24 miRNAs were identified as statistically different between HGSC with and without STIC (*q* < 0.01) but were considered biologically inconsequential in ovarian cancer given low expression of 21 out of the 24 microRNAs in the TCGA dataset. Interestingly, the number of differentially expressed miRNAs increases to 83, including additional highly expressed miRNAs, if a less stringent FDR threshold (*q* < 0.10) is applied to their miRNA dataset. Future work is needed to verify if these miRNAs represent potential unique miRNA signatures for HGSC with and without associated STIC.

We speculate that the difference between this study and Ducie *et al* 14 results from a combination of several factors including; utilization of tumor samples from multiple sites per patient, a more homogenous patient population (i.e. stage 3/4 only, exclusion of primary peritoneal diagnosis, single institution), and batch adjustment to combine separate analyses. Despite these differences, *NTS* and *LOC645591* were significantly higher in HGSC‐STIC compared to HGSC‐NOSTIC in both datasets. *LOC645591* is a noncoding transcript that is poorly annotated in the literature. BLAST analysis revealed it to be an 1193 nucleotide sequence with 99% sequence homology with the 973‐nucleotide mRNA sequence for *LHFPL3‐AS1*; an antisense transcript to the *LHFPL3* gene. *LHFPL3‐AS1*, belongs to the class of long noncoding RNAs and has been reported to be increased in metastatic melanoma 31.

*NTS*, encodes for the 13‐amino acid neuropeptide, NTS, which acts as an autocrine/paracrine signaling molecule in the nervous and gastrointestinal system. Dysregulation of NTS or one of its three receptors (NTSR1, NTSR2, NTSR3) has been reported in a variety of cancers 21, 32, 33, 34. In EOC, increased expression of NTS and its high affinity receptor NTSR1 compared to normal ovarian epithelium has been reported and overexpression of NTSR1 was associated with platinum resistance and poor prognosis 33. Conversely, NTSR3 was suggested as a potential biomarker or molecular target in HGSC based on de novo expression in tumors compared to normal OV 35. Importantly, our study is the first to compare NTS signaling in HGSC to normal FT epithelium and provides more functional evidence for its role in HGSC. We found that NTS mRNA was only increased in HGSC‐STIC compared to normal FT (see supplementary material, Figure S5). *NTS* expression was not significantly different when all HGSC samples were combined and compared to FT. Moreover, it was reported that normal ovaries express low levels of *NTS* and *NTSR1* 33. However, we observed the highest expression of *NTS* and *NTSR1* in OSE cells when compared to FTE and OvCa cells. Lastly, as opposed to *de novo* synthesis from OSE, we found evidence of increased expression of NTSR3 in FTE cells compared to OvCa cells. Taken together, our findings highlight the importance of studying HGSC in the context of normal FTE and the need to reevaluate conclusions using OSE as the normal comparison.

When co‐expressed, NTSR1 and NTSR3 interact to augment the cellular response to NTS, in part by formation of a heterodimer that is internalized following ligand stimulation 20, 36. Thus, SR48692 can inhibit the NTSR1 receptor directly or the NTSR3 receptor indirectly by preventing internalization and signal transduction. Recently, SR48692 has been proposed as a potential therapy in EOC based on increased expression of NTSR1. The present study suggests that the potential therapeutic effects of SR48692 may occur via either receptor. For example, the growth inhibitory effects of SR48692 were mimicked by NTSR3 knockdown in our study which is consistent with prior studies 35. Conversely, the migratory effects of SR48692 were mimicked by NTSR1 knockdown. Enigmatically, numerous studies have shown that NTS/NTSR1 signaling promotes cell migration. However, when co‐expressed as an NTSR1/NTSR3 heterodimer, knockdown of NTSR1 (or treatment with SR48692) may permit NTSR3 cleavage into its soluble form which stimulates cell migration 32. The unexpected observation during experimentation of NTSR1 knockdown being associated with a more epithelial phenotype while knockdown of NTSR3 resembled a more mesenchymal phenotype, is unique. Indeed, similar plasticity regarding epithelial and mesenchymal states in OvCa cells has been reported 37 and a role for NTS signaling, primarily acting through the NTSR1 receptor, in EMT has been described in several cancers 21, 38. Thus, because epithelial cells are more proliferative and exhibit greater collective cell migration, it is possible that the effects of NTSR1 knockdown reflect the shift in EMT state. Taken together, we speculate that the ratio of NTSR1/NTSR3 expression modulates the influence of NTS signaling on epithelial–mesenchymal transition in HGSC. Because of the association between EMT, peritoneal Met and poor prognosis in HGSC, future research is needed to elucidate the mechanisms involved.

Recent evidence suggests early serous proliferations may escape the FT prior to transformation into STIC 7 in a process termed ‘precursor escape’. Based on the findings of this study, we propose a model in which increased NTS signaling in HGSC‐STIC promotes epithelial to mesenchymal transition and dissemination of STIC from the FT. In support of this hypothesis, a recent study reported that increased phosphorylation of STAT3 at a site (Tyr705) which is known to be targeted by active NTS/NTSR1 signaling, is critical for HGSC progression and dissemination from STIC 39, 40.

In summary, we used a novel multisite tumor sampling approach to identify differential expression of the neuropeptide NTS between HGSC with and without STIC. Importantly, we were able to validate differential expression of *NTS* in publicly available independent datasets. This finding, along with the biological effects of NTS in OvCa cells presented herein, suggest that NTS is an important mediator in the progression and dissemination of HGSC from the FT that warrants future study.

## Author contributions statement

EJN, RNG, DLT, and MKG conceived and designed the study, processed samples, and analyzed the data. EJN, DD, and APS designed and performed in vitro experiments. CAL reviewed sample pathology and immunohistochemistry. QZ and WDJ were responsible for bioinformatics and statistical analysis. EJN wrote the manuscript and all authors edited the manuscript.

**SUPPLEMENTARY MATERIAL ONLINE**

**Supplementary materials and methods**

**Figure S1.** Small intestine was used as a positive control for NTS immunohistochemistry

**Figure S2.** Similarity matrix of paired HGSC tumors

**Figure S3.** Serum NTS levels in HGSC

**Figure S4.** Effect of RNAi knockdown of NTSR1 or NTSR3 on EMT‐associated proteins in OVCAR5 cells

**Figure S5.** Expression of mRNAs encoding NTS and its receptors in HGSC‐STIC, normal FT, and HGSC‐NOSTIC

**Table S1.** Patient characteristics

**Table S2.** List of COC samples for RNAseq analysis

**Table S3.** Normalized gene level expression (log_2_) values for each sample analyzed in this study

## Supporting information


**Supplementary materials and methods**

**Figure S1.** Small intestine was used as a positive control for NTS immunohistochemistry
**Figure S2.** Similarity matrix of paired HGSC tumors
**Figure S3.** Serum neurotensin levels in HGSC
**Figure S4.** Effect of RNAi knockdown of NTSR1 or NTSR3 on EMT‐associated proteins in OVCAR5 cells
**Figure S5.** Expression of mRNAs encoding neurotensin and its receptors in HGSC‐STIC, normal FT, and HGSC‐NOSTIC

**Table S1**. Patient characteristics

**Table S2**. List of COC samples for RNAseq analysis

**Table S3**. Normalized gene level expression (log_2_) values for each sample analyzed in this study
