# Supplementary material for: Increased expression of neurotensin in high grade serous ovarian carcinoma with evidence of serous tubal intraepithelial carcinoma
Source: J Pathol. 2019 May 14;248(3):352–62. doi: 10.1002/path.5264 (PMC6619390; doi:10.1002/path.5264)
Supplement: Supplementary file 1 — Supplementary materials and methods [file PATH-248-352-s001.docx]

**Increased expression of neurotensin in high grade serous ovarian carcinoma with evidence of serous tubal intraepithelial carcinoma**

Norris EJ *et al.* J Pathol DOI: 10.1002/path.5264

**Supplementary materials and methods**

*RNAsequencing and bioinformatics*- Next Generation RNAseq analysis was performed by Q2 Solutions-EA Genomics (Morrisville, NC, USA). For total RNAseq library construction, RNA samples were converted into cDNA libraries using the Illumina TruSeq Stranded Total RNA sample preparation kit (Illumina, San Diego, CA, USA). In brief, total RNA samples were concentration normalized to 100 ng starting input amounts, and ribosomal RNA (rRNA) was removed using biotinylated probes that selectively bind rRNA species. This process preserves mRNA and other non-coding RNA species including lncRNA, snRNA and snoRNAs. The resulting rRNA-depleted RNA was fragmented and converted into double-stranded cDNA, with dUTP utilized in place of dTTP in the second strand master mix. A single 'A' base was added to the cDNA and forked adaptors that include index, or barcode, sequences were attached via ligation. The resulting molecules were amplified via PCR. During PCR the polymerase stalls when a dUTP base is encountered in the template. Since only the second strand includes the dUTP base, this renders the first strand the only viable template, thereby preserving the strand information. Final libraries were quantified, normalized and pooled. Pooled libraries were bound to the surface of a flow cell and each bound template molecule was clonally amplified up to 1000-fold to create individual clusters. Four fluorescently labeled nucleotides were then flowed over the surface of the flow cell and incorporated into each nucleic acid chain. Each nucleotide label acts as a terminator for polymerization, thereby ensuring that a single base was added to each nascent chain during each cycle. Fluorescence was measured for each cluster during each cycle to identify the base that was added to each cluster. The dye was then enzymatically removed to allow incorporation of the next nucleotide during the next cycle.

RNAv9 pipeline:

Fluorescent signals were converted to 50 b PE reads (FASTQ files) using the standard Illumina software

(Casava). All libraries were sequenced to at least 45 M paired reads with most libraries in the 45–60 M range. The pipeline RNAv9 (EA-Quintiles) was used to analyze RNA-seq data. To prepare the reads for alignment, the sequencing adapters and other low quality bases were clipped. A subset of the reads (~1 million reads) were aligned to spiked-in control sequences (PhiX and other Illumina controls used during library preparation), residual sequences (globin and ribosomal RNA), and poly-A/T sequences that persisted after clipping. The reads were also aligned to a sampling of intergenic regions to assess DNA contamination level and set the RPK (reads per thousand bases) threshold to call a transcript as present. STAR was used as the alignment tool, while RSEM was used to quantify genes and transcripts. The RSEM v1.2.0 program, rsem-calculate-expression, was run with parameters optimized for Illumina 50x50 paired-end sequencing. The UCSC known gene transcriptome was used. For across sample analysis, upper-quartile normalization of the read counts was performed.

*Assignment of tumor origin in TCGA dataset*- Ovarian cancer RNAseq data and pathology reports were downloaded from [www.cbioportal.org.](http://www.cbioportal.org/) Pathology reports were randomly selected from patients with stage III or IV HGSC and reviewed for assignment as either FT-HGSC or O-HGSC. Patients were assigned as FT-HGSC if the pathology report included a diagnosis of Fallopian tube carcinoma or included any of the following terms: “serous tubal intraepithelial carcinoma”, “in situ carcinoma” in the Fallopian tube epithelium, or “intramucosal” Fallopian tube involvement. Patients were assigned as OHGSC if the pathology report stated that a STIC was not identified, the tubal mucosal was benign, or if microscopic examination of serial sections of the Fallopian tube revealed no evidence of tumor involvement.

*Cell culture conditions*- Immortalized Fallopian tube cells were maintained in 1:1 DMEM/Ham’s F12

Media supplemented with 2 mM glutamine, 2 % Ultroser™ g (Pall Corporation, Port Washington, NY, USA) and 1000 units/mL Penicillin and Streptomycin. HOSE cells were maintained in 1:1 DMEM/Ham’s F12 Medium supplemented with 2 mM glutamine and 10% fetal bovine serum (FBS) (Atlantic Biologicals, Miami, FL, USA). IOSE cells were maintained in 1:1 Medium 199/MCDB Medium 105 (Sigma Aldrich, St. Louis, MO, USA) with 2 mM glutamine and 10% FBS. OvCa lines were maintained in RPMI 1640 with 10% FBS and 2mM glutamine. All cells were passaged 2-3 times per week to maintain logarithmically growing cultures. All cell culture reagents were purchased from ThermoFisher Scientific (Waltham, MA, USA) unless otherwise stated.

*RT-qPCR*- Total RNA (0.5 µg) was reverse transcribed into cDNA using the Superscript Vilo IV Master Mix (ThermoFisher Scientific) and diluted with DEPC-treated water to a final concentration of 20 ng/µl. Realtime qPCR was performed in triplicate in 20 µl final volumes using 40 ng of cDNA and Taqman Assay Reagents and multiplexed with a reference gene (*UBTF*). Levels of *NTS*, *NTSR1*, *NTSR2*, and *NTSR3* were normalized to *UBTF* which we previously identified as invariant in HGSC tumor samples and OvCa cell lines. All procedures were performed according to the manufacturer’s protocol. The Taqman Assay IDs were Hs00175048_m1 (*NTS*), Hs00901551_m1 (*NTSR1*), Hs00892563_m1 (*NTSR2*), Hs00361760_m1 (*NTSR3*), and Hs01115792_g1 (*UBTF*).

*Western blotting-* Cells were lysed using Radioimmunoprecipation Assay (RIPA) buffer and centrifuged at 100,000 x *g* for 1 h at 4 °C. Supernatant was collected and protein concentration was determined using the Bradford Assay (Biorad, Hercules, CA, USA). Twenty micrograms of protein were separated by SDS-PAGE according to the BOLT™ western blotting protocol (ThermoFisher Scientific). Proteins were transferred to PVDF membranes and blocked with 5% nonfat dry milk in TBS/0.05% TWEEN 20 buffer for 1 h at room temperature. Membranes were incubated with primary antibodies against NTSR1 (SAB4300718, 1:2500, Sigma Aldrich, St. Louis, MO), NTSR3 (ab16640, 1:1000, Abcam, Cambridge, UK), β-Actin (clone AC-15, 1:40,000, Sigma Aldrich), E-Cadherin (1:1000, 24E10, Cell Signaling Technology, Danvers, MA), N-Cadherin (1:1000, D4R1H, Cell Signaling Technology) and β-Catenin (1:1000, D10A8, Cell Signaling Technology) overnight at 4º, washed 3x, and incubated for 1 h at room temperature with appropriate secondary antibody. Proteins were visualized using Amersham ECL Prime (GE Healthcare Life Sciences, Marlborough, MA) and imaged using UVP GelDoc-it Imaging Station and software (UVP, Jena, Germany).

ELISA- A commercially available competitive enzyme immunoassay (EIA) against human neurotensin was used to determine serum NTS levels according to manufacturer’s protocol (EIA-NT-1, Raybiotech, Norcross, GA, USA).
