# Supplementary material for: Increased expression of neurotensin in high grade serous ovarian carcinoma with evidence of serous tubal intraepithelial carcinoma
Source: J Pathol. 2019 May 14;248(3):352–62. doi: 10.1002/path.5264 (PMC6619390; doi:10.1002/path.5264)
Supplement: Supplementary file 2 — Figure S1. Small intestine was used as a positive control for NTS immunohistochemistry Figure S2. Similarity matrix of paired HGSC tumors Figure S3. Serum neurotensin levels in HGSC Figure S4. Effect of RNAi knockdown of NTSR1 or NTSR3 on EMT‐associated proteins in OVCAR5 cells Figure S5. Expression of mRNAs encoding neurotensin and its receptors in HGSC‐STIC, normal FT, and HGSC‐NOSTIC [file PATH-248-352-s002.docx]

**Increased expression of neurotensin in high grade serous ovarian carcinoma with evidence of serous tubal intraepithelial carcinoma**

Norris EJ *et al*. *J Pathol* DOI: 10.1002/path.5264


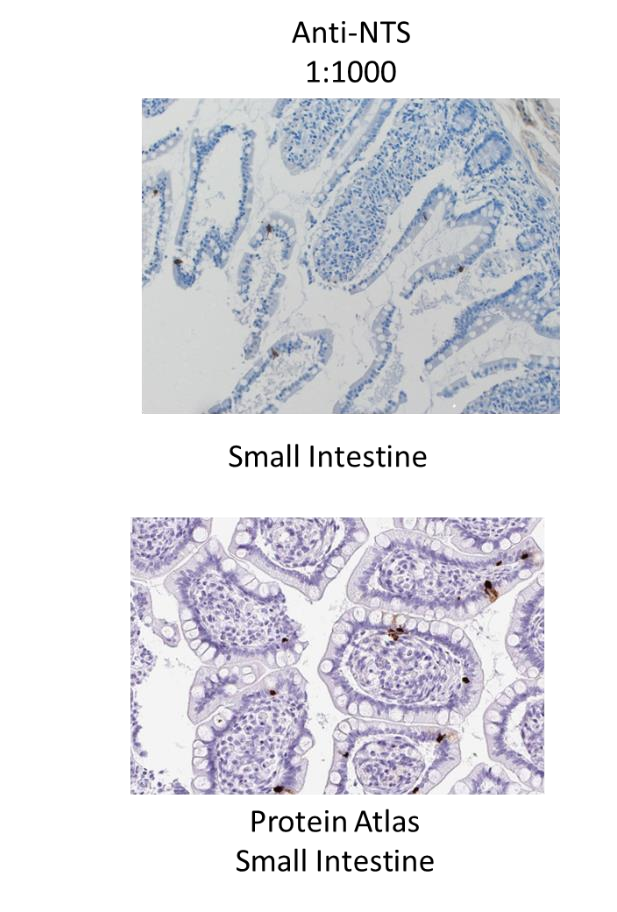


**Figure S1.** Small intestine was used as a positive control for NTS immunohistochemistry. Staining of small intestine using our staining protocol (top: 1:1000,#AB5496, EMD Millipore, Burlington, MA, USA) resembled NTS staining (Antibody HPA026664, Sigma Aldrich, St. Louis, MO) found in the ProteinAtlas (bottom) available at:
https://www.proteinatlas.org/ENSG00000133636-NTS/tissue/small+intestine#imid_15402488


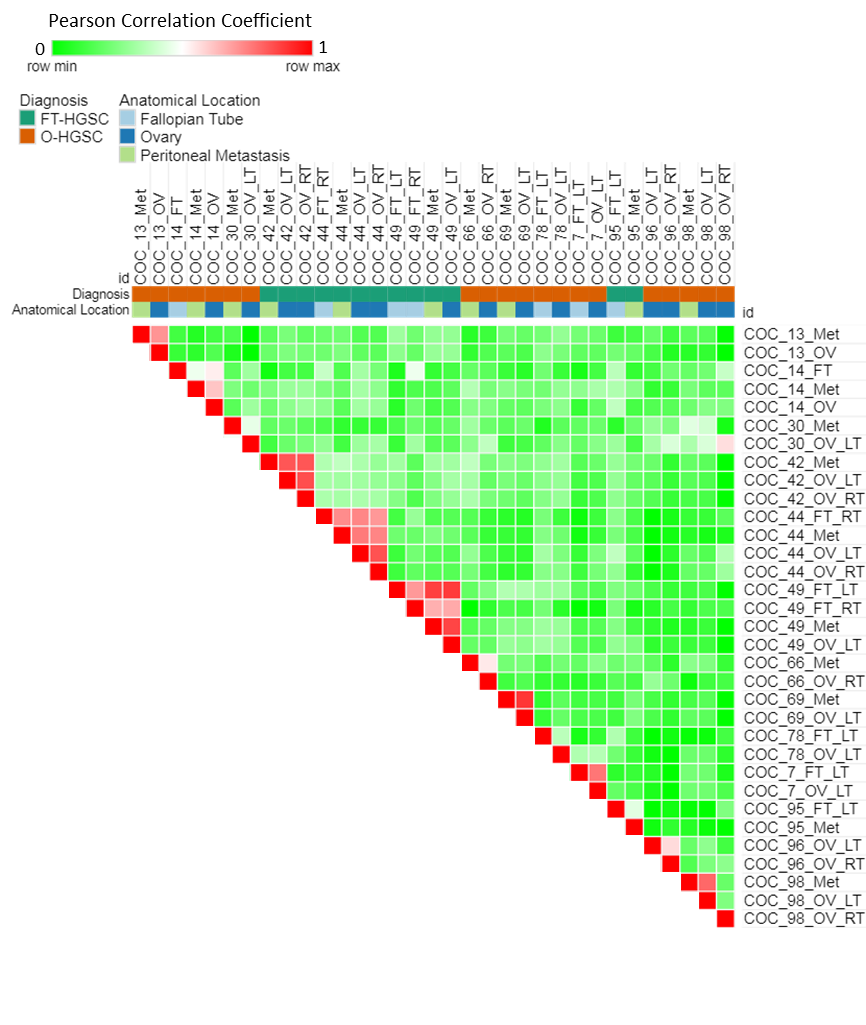


**Figure S2.** Similarity matrix of paired HGSC tumors.

A similarity matrix was constructed using the Pearson Coefficient of Correlation between all samples from patients with paired tumors to quantify the degree of inter- and intra-patient tumor heterogeneity. In total, 33 samples were compared from 13 different patients. Green indicates low correlation, whereas red indicates a high degree of correlation as noted. Diagnosis (presumed site of tumor origin) and the anatomical location where each tumor sample was collected color coded as indicated.

P=0.0165

HGSC

**Figure S3.** Serum neurotensin levels in HGSC.

Serum neurotensin levels were analyzed using a commercially available enzyme immunoassay specific for human neurotensin. Serum was collected from 15 patients with HGSC and 9 patients undergoing surgery for benign conditions. A two-tailed Student’s *t* test was used to test for statistical significance (P<0.05)

Actin

SNAIL

E-cadherin

N cadherin


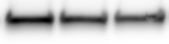

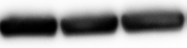


Non-Silencing RNAi

RNAi NTSR1

RNAi NTSR3

OVCAR5

**Figure S4.** Effect of RNAi knockdown of NTSR1 or NTSR3 on EMT-associated proteins in OVCAR5 cells. Protein lysates were prepared from OVCAR5 cells treated for 96 h with RNAi targeting *NTSR1* or *NTSR3*. A non-silencing RNAi served as a control. Levels of E-cadherin, N-cadherin, and SNAIL were assessed using commercially available antibodies. β-actin served as a loading control. The western blot is representative of three individual experiments.

#


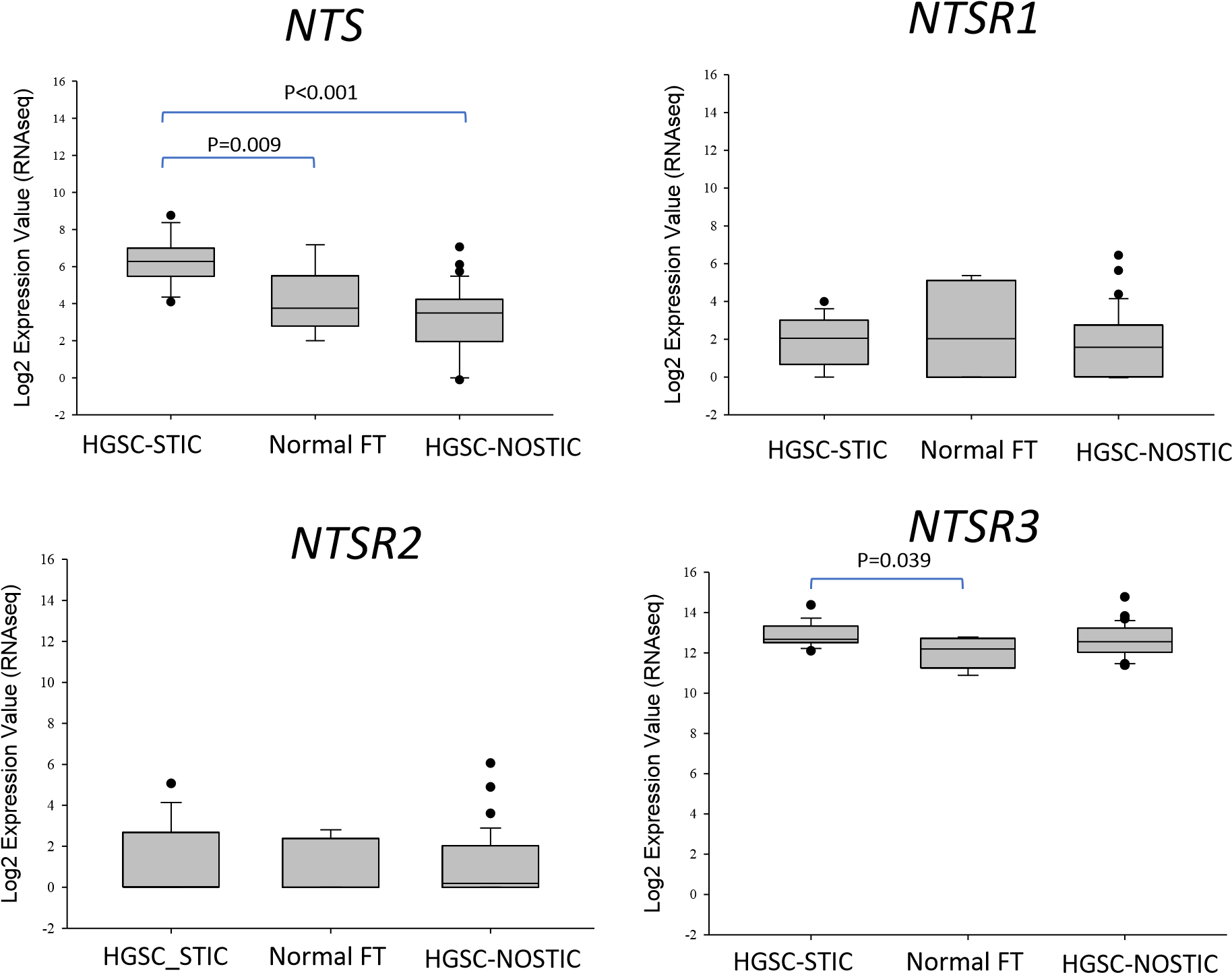


**Figure S5.** Expression of mRNAs encoding neurotensin and its receptors in HGSC-STIC, normal Fallopian tube, and HGSC-NOSTIC.
