# Supplementary material for: Increased expression of neurotensin in high grade serous ovarian carcinoma with evidence of serous tubal intraepithelial carcinoma
Source: J Pathol. 2019 May 14;248(3):352–62. doi: 10.1002/path.5264 (PMC6619390; doi:10.1002/path.5264)
Supplement: Supplementary file 3 — Table S1. Patient characteristics [file PATH-248-352-s003.docx]

**Increased expression of neurotensin in high grade serous ovarian carcinoma with evidence of serous tubal intraepithelial carcinoma**

Norris EJ *et al.* J Pathol DOI: 10.1002/path.5264

# **Table S1.** Patient characteristics

|  |  | OV-HGSC (N=15) | FT-HGSC (N=9) | P value |
| --- | --- | --- | --- | --- |
| Mean age at surgery (years; range) |  | 63 (43–82) | 64 (53–80) | 0.133 |
| Stage | III | 15 | 9 |  |
|  | IV | 0 | 0 |  |
| CA125 U/ml (range) |  | 877 (11-3353) | 635 (133-3617) | 0.361 |
| Menopausal status | Pre-menopause | 3 | 1 |  |
|  | Post-menopause | 12 | 8 |  |
